# Association of characteristics of delivery and medical conditions during the first month of life with developmental defects of enamel

**DOI:** 10.1186/1472-6831-14-122

**Published:** 2014-10-01

**Authors:** Mahtab Memarpour, Ali Golkari, Reihaneh Ahmadian

**Affiliations:** Department of Pediatric Dentistry, School of Dentistry, Shiraz University of Medical Sciences, Shiraz, Iran; Department of Dental Public Health, School of Dentistry, Shiraz University of Medical Sciences, Shiraz, Iran; Student Research Committee, School of Dentistry, International Branch, Shiraz University of Medical Sciences, Shiraz, Iran

**Keywords:** Dental enamel, Infant, Apgar score, Birth order, Permanent teeth

## Abstract

**Background:**

Developmental defects of enamel (DDE) may be influenced by health problems and illness in children. The aim of the study was to identify the main characteristics of birth (delivery) and adverse medical conditions during the first month of life that may be related to DDE in permanent teeth.

**Methods:**

1000 schoolchildren between the ages of 9 and 11 years were selected for this cross-sectional study by multistage randomization from four educational zones in Shiraz in 2013. Intraoral examination was used to diagnose DDE according to World Health Organization screening guidelines and the Modified DDE Index. The data on seven birth factors as well as health and nutritional conditions during the first month of life were collected by a questionnaire completed by the parents, and were analyzed with the chi-squared test, Spearman’s correlation and binary logistic regression.

**Results:**

469 (48.2%) out of 974 schoolchildren had at least one permanent tooth with DDE. The defects were significantly related with Apgar score at birth <7 (p = 0.003) and illness during the first month (p = 0.035). The frequency of DDE was significantly lower in the third child in families compared to the first and second child (p = 0.005). However, DDE showed no significant relationship with gestational age, delivery type, birth weight, gender or type of feeding during early infancy.

**Conclusions:**

Three associated factors were identified (birth Apgar score, illness during the first month of life, birth order) for DDE in permanent teeth. No specific illness was found to be significantly associated with DDE.

## Background

Tooth enamel may be affected by factors that occur before or after tooth eruption or during tooth development. The factors that occur during tooth development are caused by disturbances in the formation and calcification phases of tooth enamel development, and can lead to developmental defects of enamel (DDE) before tooth eruption [1]. These defects are divided in two main groups: hypomineralization (change in the quality of the enamel) and hypoplasia (change in the quantity). In hypomineralization, changes in enamel transparency are visible as staining or opacity.

Epidemiological studies have reported that the prevalence of DDE ranges widely between 23% and 49% in primary teeth and between 18% and 63% in permanent teeth [2–11]. DDE may cause problems for children such as increased tooth sensitivity, poor appearance [11, 12], increased risk of dental caries [7, 13–18] and dental attrition [19].

DDE may develop because of factors that affect the formation or secretion of enamel. Factors associated with DDE can be categorized as localized or generalized, and generalized factors can be further divided into environmental and hereditary, both of which influence enamel during prenatal, neonatal and postnatal development [20]. Chemical elements and medicines administered to young children or their mothers during pregnancy have been cited as other important factors [21, 22]. Among these factors, some occur before birth or during the first months of infancy, and can affect children’s general health as well as their future oral and dental health. Earlier studies reported that low birth weight is associated with an increased frequency of enamel hypoplasia and DDE in primary teeth [23, 24]. Also some conditions may cause DDE or molar-incisor hypomineralization in permanent teeth [25, 26]. In addition, low Apgar score is a high risk factor for dental caries in preschoolchildren [27]. Apgar score is a rapid method to evaluate the clinical status of newborn infants [28]. Research has shown the DDE in primary teeth may be related with a low Apgar score, inadequate parenteral nutrition or multiple births [29].

Medical problems during the perinatal and postnatal periods are other factors that can lead to the development of DDE, in addition to those identified in previous studies [30, 31]. A history of infectious or congenital diseases in children is a predisposing factor for enamel hypoplasia or DDE in the primary or permanent teeth [7, 32, 33]. Most of these diseases and medical conditions appear to act not directly on the ameloblasts, but by affecting the child’s nutrition and growth; as a result, ameloblasts do not obtain the nutrients they need to secrete enamel.

Because DDE occur due to changes during tooth development and cause problems for patients, it is important to identify adverse conditions during tooth formation. However, most studies have been designed to discover associations between prenatal and perinatal conditions and DDE, molar-incisor hypomineralization or enamel hypoplasia in primary teeth [22–24, 30, 31]. Only a few studies have considered the permanent dentition [25, 26], and most of them have considered specific diseases only [33]. In an effort to consider a variety of adverse conditions that can affect children at a specific period of time and that may be factors associated with DDE in the permanent dentition, this study investigated the effect of birth characteristics and adverse medical conditions during the first month of life on the presence of DDEs in permanent teeth in a representative group of 9- to 11-year-old children.

## Methods

This retrospective study was designed and conducted during the 2013 school year, and the participants were 9- to 11-year-old children who were attending primary schools in Shiraz. Ethical approval was obtained from Shiraz University of Medical Sciences and the Shiraz Educational Head Office (Grant no. 8591024). The children were selected by multistage randomized sampling. The city was divided geographically into four areas based on the zones established by the Educational Head Office. Then four primary schools were randomly selected in each zone (two girls’ schools and two boys’ schools, as schools are segregated according to gender in Iran). At each school 80 pupils were selected randomly in the fourth and fifth grades. If the total number of fourth and fifth graders at a given school was less than 80, all students were included, and if the total number was more than 80, students were selected randomly by using simple randomization. The main inclusion criterion was age between 9 years, 0 months and 11 years, 11 months. All children had resided in Shiraz city since birth to the time of study, and the level of fluoride in drinking water was approximately the same (0.3-0.7 ppm) throughout the city. The exclusion criteria were systemic or chronic diseases such as heart disease, congenital physical or mental disability, full-coverage restoration in a permanent tooth, filling or fissure sealant on the buccal or palatal surfaces, orthodontic brackets, lack of parental consent to participate in research and lack of cooperation with the examination. Also questionnaires that were not returned or in which more than one item was left blank were excluded from the study.

A specially-designed questionnaire for the parents was developed. The aims and procedures of the study were explained and participants’ privacy was ensured in the consent form. The parents were asked to complete and return the questionnaire only if they consented to their child’s inclusion in the study. A phone number was provided in case they needed more information about the study or the questionnaire. Parents who provided their consent for their child to participate were asked to complete the questionnaire. The items on birth conditions were based on the Health Status Evaluation Form that must be completed and filed by the attending doctor or midwife at birth. The parents were asked to refer to their children’s birth record when answering these questions. The data were collected in two parts:

1. Demographics (date of birth, gender, place of residence since birth), type of delivery (natural, aided or cesarean), birth weight (less than 2500, between 2500 and 4000, and more than 4000 grams), gestational age (less than 38, between 38 and 42, and more than 42 weeks), birth Apgar score (less than 7 and 7 or more) [28], birth order (first child, second child, or third/later child), number of babies delivered (singleton, twins or triplets) and mother’s age at delivery (less than 20, between 20 and 35, and more than 35 years). In the second part of the questionnaire, the parents were asked about the type of infant feeding, name and duration of diseases (if any), and hospitalizations (if any) during the first month of life. This part of the questionnaire was completed by the parents.
2. DDE were recorded by the project investigators during intraoral examinations.

After explaining the purposes of the study to the schoolchildren, information on oral health education was provided in groups. Each selected child who returned the signed consent form and questionnaire was given a toothbrush and toothpaste and asked to clean his/her teeth under supervision. Intraoral examination was performed by two senior dental students (after appropriate calibration) to record DDE. The examiners’ evaluations were calibrated by having them examine 20 patients under supervision by the professor. Intra-examiner reliability was checked during the calibration sessions, and inter-examiner reliability was alpha = 0.73. The examiner first cleaned the teeth with sterile gauze, then examined all the permanent teeth with a disposable mirror, explorer and head light. The criteria for DDE were based on the instructions and oral health screening forms provided by the World Health Organization (1997) and the modified DDE index for screening studies (1987) [34]. In the present study, the number of children with at least one DDE was recorded. In addition the mean number of the teeth with DDE was calculated for the whole sample of children. The examinations were supervised by two faculty members with extensive experience in this subject, who also participated in the calibration process.

The SPSS (version PASW 18; IBM, USA) was used for data entry and analysis. The percentage of children with at least one DDE in a permanent tooth and the average number of permanent teeth with a DDE per child were calculated. The relationship between having a DDE in a permanent tooth and each of the characteristics of birth (type of delivery, birth weight, gestational age, birth Apgar score, birth order, number of babies delivered and mother’s age at delivery) and the first month of infancy (feeding in the first months and illness in the first months) was analyzed with the chi-squared test. The relationships were assessed once again after adjustments. For this purpose, along with sex and number of permanent teeth present, all variables with a bivariate p value less than 0.250 were entered in a binary logistic regression model to adjust for the effects of possible confounders. Spearman’s correlation test was used to identify correlations between the duration (in weeks) of illness or hospitalization and the number of permanent teeth with DDE.

## Results

A total of 974 (97%) children (average age ± standard deviation: 10.23 ± 0.66 years) who met the inclusion criteria participated in the study and returned their parents’ completed questionnaire; 484 (49.7%) were boys and 490 (50.7%) were girls. 341 of the children attended a private school and the rest were from public schools (Table 1). Fourteen restored permanent teeth were excluded from the study; however these children were not excluded from the analysis. Ten children were excluded because their parents did not provide their informed consent, six were excluded because of blank items in their returned questionnaires, and four children were absent on the days of both visits to their school.

**Table 1:** **Number of children by sex, school type and school zone**

Sex	Zone	School type, total N (N with DDE,% with DDE)		Total
		Public	Private	
**Boys**	1	80 (34, 42.5%)	39 (16, 43.6%)	119 (51, 42.9%)
	2	80 (41, 51.3%)	48 (22, 45.8%)	128 (63, 49.2%)
	3	73 (51, 69.9%)	41 (15, 36.6%)	114 (66, 57.9%)
	4	80 (23, 28.8%)	43 (22, 51.2%)	123 (45, 36.6%)
	All	313 (149, 47.6%)	171 (76, 44.4%)	484 (225, 46.5%)
**Girls**	1	80 (36, 45.0%)	50 (26, 52.0%)	130 (62, 47.7%)
	2	80 (41, 51.3%)	42 (25, 59.5%)	122 (66, 54.1%)
	3	80 (24, 30.0%)	39 (12, 30.8%)	119 (36, 30.3%)
	4	80 (57, 71.3%)	39 (23, 59.0%)	119 (80, 67.3%)
	All	320 (158, 49.4%)	170 (86, 50.6%)	490 (244, 49.8%)
**Total**		633 (307, 48.5%)	341 (162, 47.5%)	974 (469, 48.2%)

In parentheses, the percentage of children with DDE in each group.

In all, 469 children (48.2%) had at least one tooth with DDE. The percentage of boys (46.5%) and girls (49.8%) with DDE was similar, as was the percentage of children with DDE from public (48.5%) and private schools (47.5%). The average number of teeth with DDE per child was 2.13 ± 3.48. The difference among the four educational zones in the average number of teeth with DDE (range 1.9 to 2.13) was not statistically significant (p = 0.589). Neither was this average significantly different between sexes (p = 0.239) or between children from public and private schools (p = 0.778).

Sixty-one (6.3%) children had a birth Apgar score less than 7. Among them, 39 (63.9%) had at least one DDE in a permanent tooth; the rate of DDE among those with a birth Apgar scored of 7 or more (913 children) was 47.1%. The difference between the two groups was statistically significant (p = 0.003), i.e., children born with an Apgar score of less than 7 were more likely to have DDE in their permanent dentition. Just below 50% of the children were the firstborn child in their family. About a quarter of the children were the second child, and the other quarter of children were the third child (or later) in the family. The percentage of DDE in their permanent teeth was 50.3% among firstborn children, 51.7% among second children, and 40.1% among children who were third or later in birth order. Thus, children in the third group were significantly less likely to have a DDE in their permanent teeth than the other two groups (p = 0.005). No other birth characteristic and no type of feeding was significantly related with the frequency of DDE in the permanent teeth (Table 2).

**Table 2:** **Relationship between DDE in permanent teeth and birth conditions, type of feeding and illness in the first month of life**

Factor	Groups	Number of children in group	Number of children with DDE	Percentage of children with DDE	p-value before any adjustment
**Delivery type**	Natural	506	247	48.8	
	Aided	11	8	72.7	0.182
	Cesarean	414	191	46.1	
**Birth weight (g)**	< 2500	94	42	44.7	
	2500-4000	763	367	48.1	0.639
	> 4000	59	30	50.8	
**Gestational age (weeks)**	< 38	308	151	49.0	
	38-42	489	232	47.4	0.842
	> 42	21	11	52.4	
**Birth Apgar score**	< 7	61	39	63.9	0.011
	≥7	913	430	47.1	
**Birth order**	1st	453	228	50.3	
	2nd	240	124	51.7	0.016
	3rd or later	237	95	40.1	
**Number of newborns deliveries**	Single	953	458	48.1	0.539
	Multiple (Twins or triplets)	20	11	55.0	
**Mother’s age at delivery (years)**	< 20	167	79	47.3	
	20-35	715	340	47.6	0.948
	>35	44	22	50.0	
**Feeding in the first month**	Breast milk	833	394	47.3	
	Formula	37	17	45.9	0.429
	Both	61	34	55.7	
**Illness in the first month**	Yes	144	81	56.3	0.035
	No	830	388	46.7	

The parents of 144 children (14.8% of the total) reported that their child had a serious illness between birth and 1 month of age. Of this group, 81 children (56.3%) had at least one permanent tooth with DDE. As shown in Table 2, this rate was significantly higher than the 46.7% rate in children with no reported illnesses during their first month of life (p = 0.035). No specific disease was found to be more (or less) likely to be related with the presence of DDE. However, the number of DDE in the permanent teeth increased with the duration (in weeks) of the illness (r = 0.423, p = 0.006).

As seen in Table 3, all factors found in the bivariate analysis to be significantly related with the likelihood of having a DDE in permanent teeth remained significant after adjustment. The regression models showed that children with an Apgar score of less than 7 were 2.32 times more likely than the others to develop a DDE (OR = 2.32, 95% CI: 1.31-4.11, p = 0.004). Children who were their mothers’ third child or later in birth order were significantly less likely to develop a DDE than firstborn (p = 0.007) or secondborn children (p = 0.006). Illness in the first month of life also remained significantly associated with having a DDE after adjustment (OR = 1.58, 95% CI: 1.08-2.31, p = 0.017). Moreover, regression analysis showed that girls were 1.33 times (95% CI: 1.02-1.74, p = 0.037) more likely to have a DDE than boys. The number of permanent teeth present showed no significant association with the risk of having a DDE. This was probably due to the fact that the permanent incisors and first molars had erupted in all children included in the present study. Therefore, if there had been a risk of having a DDE the lesion was most likely to appear in these teeth rather than in the premolars or canines.

**Table 3:** **Association between DDE in permanent teeth and variables with a p-value less than 0.250 in the bivariate analysis, plus sex and the number of permanent teeth present**

		Odds ratio (95% CI)	p-value
**Sex**	Boy	1	-
	Girl	1.33 (1.02-1.74)	0.037
**Number of permanent teeth**		0.98 (0.95-1.01)	0.152
**Delivery type**	Natural	1	-
	Aided	2.77 (0.69-10.86)	0.152
	Cesarean	0.90 (0.69-1.17)	0.423
**Birth Apgar score**	≥7	1	-
	<7	2.32 (1.31-4.11)	0.004
**Birth order**	1st	1.57 (1.13-2.17)	0.007
	2nd	1.68 (1.16-2.45)	0.006
	3rd or more	1	-
**Illness in the first month**	No	1	-
	Yes	1.58 (1.08-2.31)	0.017

## Discussion

This study investigated the effects of factors during delivery and the first month of life on the subsequent appearance of DDE in the permanent teeth of children in the 4th and 5th grades of primary school in Shiraz. The overall prevalence of DDE was 48.2%, which was close to the figures reported by others [3, 10, 11]. We selected this age group because in most children their permanent incisors and first molars had erupted, and most of the newly erupted teeth were not affected by attrition or erosion and therefore would show DDE more clearly than aged teeth.

The number of boys and girls was almost equal. The numbers of children from different educational zones were also similar. However, the number of children from private schools was lower than those from public schools. Although the percentage of boys and girls with a DDE in school zones 3 and 4 might have differed, the two areas offset each other and therefore no significant difference was seen between all boys and all girls in our sample.

The secretion phase of enamel development in the permanent dentition starts around birth [1]. The circumstances of birth and the first month of life are therefore important both as the starting point for enamel secretion and as periods of life that affect the child’s nutrition and growth during subsequent years. Most previous studies of the risk factors for DDE analyzed the direct effects of illness and other adverse conditions. The “direct effects” of each associated factor are expected to affect the enamel that is being formed at the time when each factor occurred. The formation of most permanent teeth had not yet started during the age range (birth to 1 month) we investigated [35]. Nevertheless, “indirect effects” are also important to consider. In other words, birth and perinatal factors would be expected to affect the child’s general health in later life [36]. Accordingly, DDE in the permanent dentition can be used as a marker of a child’s general previous health status.

Apgar score is used to evaluate the infant’s general health immediately after delivery [37]. We found that a low birth Apgar score may be related with DDE in the permanent teeth. The same result was reported earlier for primary teeth [29, 37]. Among the factors that may affect Apgar score are gestational age, maternal medication, resuscitation, cardiorespiratory and neurologic conditions, infections, hypoxia, hypovolemia and preterm delivery [28].

Like other researchers, we found that DDE were more frequent in children with a history of illness during the first month of life than in children without this antecedent. Children with a history of respiratory infection, chickenpox congenital rubella or otitis reportedly had more in their primary teeth and permanent teeth [7, 32, 33] than their peers without a history of these illnesses. Such illnesses may lead to hypocalcemia, hypoxia and pyrexia in the child or the mother [30]. This may be related with the effect of illness on enamel formation, or may reflect the influence of drugs such as amoxicillin, which can indirectly affect enamel [38].

We found no significant relationship between DDE and low birth weight or preterm delivery. Some studies reported these factors to be associated with DDE in the primary teeth [23, 24, 29] or permanent teeth [25, 26]. Their effect on primary dentition may be related to the fact that most enamel in the primary teeth is secreted before birth, so an adverse condition during gestation might affect birth weight and lead to DDE in the primary dentition.

The differences between our results and factors previously associated with DDE in the permanent teeth may be related to differences in the categories used to record low birth weight or gestational age [25, 27, 39]. For example, some researchers defined preterm delivery as a gestational age ≤36 weeks, and defined low birth weight as <1500 g. In addition, the low numbers of children in some of the categories we used, such as low or high birth weight, instrumental delivery and multiple deliveries, may be another reason for the absence of significant differences between groups. For example, 8 out of 11 children with instrumental delivery had DDE, and the percentage frequency of 72.7% with DDE in this subgroup was much higher than the 48.2% average. The low number of children in this category thus probably accounted for the absence of significant differences compared to other subgroups.

We found that birth order was related with the prevalence of DDE in our sample. Third children had significantly fewer DDE than firstborn children, a finding that may be related to low birth weight, mother’s age during pregnancy or nutritional deficiency [40, 41].

The response rate was about 98%, which was relatively high considering the nature of the study. The results of this study can be assumed to be essentially unaffected by the data we excluded from analysis. A limitation of the present study was the use of a self-completed questionnaire to obtain information about circumstances during delivery and the first month of life. Birth conditions were recorded in the children’s birth record, which is mandatory in Iran. These items in the questionnaire thus probably reflected accurate information. However, parents may not accurately recall events during the child’s first months of life or may not have access to their child’s perinatal medical record. The questionnaire items that depended on parents’ recall may thus have been influenced by the variable reliability of the information our respondents provided. To overcome this limitation a cohort study would be needed, based on accurate childbirth and follow-up records in addition to information about the frequency of DDE.

## Conclusions

This study found a prevalence of DDE in permanent teeth of 48.2% in children aged 9 to 11 years. There was a significant relationship between DDE and three early life factors that may affect tooth formation by having long-term effects on general health. A birth Apgar score less than 7 and illness during the first month of life had negative effects on enamel development. The duration of illness was more important than the specific type of illness. In addition, being the third child (or later) in a family was significantly related with a lower rate of DDE in comparison to being the firstborn child.

## Abbreviations

DDE: Developmental defects of enamel.
